# Deliberative dialogues in rural South Africa reveal public support and communication challenges in HIV cure research: uMkhanyakude district, KwaZulu-Natal

**DOI:** 10.1093/inthealth/ihaf131

**Published:** 2025-11-14

**Authors:** Rujeko Samanthia Chimukuche, Miliswa Magongo, Qinisile Shandu, Nomathamsanqa Majozi, Ingrid V Bassett, Janet Seeley, Thumbi Ndung’u

**Affiliations:** Africa Health Research Institute, Social Science Unit, KwaZulu-Natal, 4013, Durban, South Africa; Division of Infection & Immunity, University College London, WC1E 6BT, London, UK; Africa Health Research Institute, Social Science Unit, KwaZulu-Natal, 4013, Durban, South Africa; Africa Health Research Institute, Social Science Unit, KwaZulu-Natal, 4013, Durban, South Africa; Africa Health Research Institute, Social Science Unit, KwaZulu-Natal, 4013, Durban, South Africa; Africa Health Research Institute, Social Science Unit, KwaZulu-Natal, 4013, Durban, South Africa; Division of Infectious Diseases, Massachusetts General Hospital, 02114, Boston, MA, USA; Africa Health Research Institute, Social Science Unit, KwaZulu-Natal, 4013, Durban, South Africa; School of Nursing and Public Health, University of KwaZulu-Natal, 4041, Durban, KwaZulu-Natal, South Africa; Department of Global Health and Development, London School of Tropical Hygiene and Medicine, WC1E 7HT, London, UK; Africa Health Research Institute, Social Science Unit, KwaZulu-Natal, 4013, Durban, South Africa; Division of Infection & Immunity, University College London, WC1E 6BT, London, UK; HIV Pathogenesis Programme, The Doris Duke Medical Research Institute, University of KwaZulu-Natal, 4013, Durban, South Africa; Ragon Institute of Massachusetts General Hospital, Massachusetts Institute of Technology and Harvard University, Cambridge, 02139, MA, USA

**Keywords:** community engagement, deliberative methods, HIV cure, social science and HIV cure

## Abstract

**Background:**

There is a need to build public support for the conduct of HIV cure research. It is important to enhance the public understanding of HIV cure strategies in resource-constrained settings with high HIV prevalence. Adopting deliberative approaches allows information to be shared interactively, building knowledge on the topic and unpacking the complexity of the science.

**Methods:**

We conducted deliberative community dialogues in rural KwaZulu-Natal, South Africa, with the Africa Health Research Institute community engagement team and Community Advisory Board members. We discussed the public understanding of ‘HIV cure’ and current scientific approaches. Detailed written notes were taken in anonymised format. Data were later analysed thematically, with three main themes identified: (i) understanding of HIV cure trials; (ii) HIV scientific procedures; and (iii) HIV cure messaging.

**Results:**

Participants’ questions and discussion revealed support for research on HIV cure but limited knowledge and understanding. Participants had questions about trial procedures, risks of antiretroviral therapy interruptions, efficacy, cost and monitoring of viral loads. There is need for simple, accessible language about the complex science to avoid misunderstanding among community members.

**Conclusion:**

On going challenges in effectively communicating complex scientific concepts highlight the importance of ongoing community engagement and education in HIV cure research.

## Introduction

In 2023, 40 million people were living with HIV and 1.3 million people newly acquired HIV.^1^ The rising numbers of people living with HIV (PLWH), because people are living longer on antiretroviral therapy (ART) and falling HIV incidence, reflect the tremendous scientific and public health success in the global response to HIV, which has resulted in reduced morbidity and mortality for PLWH.^2^ However, recent geopolitical events and realignment of priorities by key funders now threaten the progress in HIV prevention and care services, particularly in low- and middle-income countries (LMICs), and it is projected that there will be an increase in the number of people acquiring HIV.^3,4^ In PLWH who are not on ART the virus replicates, leading to advanced HIV-related disease and death in most cases. However, for PLWH who are adherent to ART, plasma virus levels are suppressed and maintained at undetectable levels, which significantly reduces HIV-associated morbidity and mortality and prevents transmission of the virus. Although ART is highly beneficial, it does not cure HIV infection and, if treatment is stopped, the virus rebounds within weeks due to the persistence of a reservoir of latently infected cells that are difficult to eradicate.^5–7^ As we anticipate increasing numbers of PLWH, an HIV cure is needed urgently. An HIV cure would not only address the challenges of new infections and the health burden associated with taking ART for life, it would ultimately eliminate HIV as a global health problem.^8^

### The science of HIV cure

Based on observations that HIV can be completely eradicated from the body and that it can also be durably controlled in the absence of ART, a scientific consensus has emerged that eradication or durable ART-free control (remission) should be the two main goals of HIV cure strategies.^8,9^ Virus eradication is still considered the ultimate goal in HIV cure research and is the preference of most PLWH,^10^ but, to date, it has only been achieved in patients undergoing clinically risky and expensive allogeneic stem cell transplantation for life-threatening haematological malignancies.^11^ Viral eradication is therefore currently not a viable public health strategy unless less invasive and cost-effective gene therapy approaches can be developed. By contrast, cases of natural control of HIV in the absence of any ART intervention, or remission following ART interruption, are more common, and although the mechanisms appear to be diverse and not yet fully understood,^12–14^ there is hope that this approach may be more widely applicable to communities in great need, particularly through combination immunotherapeutic interventions.^9^

To date, HIV cure research (particularly clinical trials) has largely been conducted in high income settings^15^ with limited research conducted in urban settings in LMICs. For example, a recent phase 2a HIV cure trial (NCT05281510) at the Females Rising through Education, Support and Health (FRESH) site in Durban, South Africa, enrolled 20 women participants who initiated ART during acute HIV, and assessed the safety and efficacy of two broadly neutralising antibodies (VRC07-523LS and CAP256V2LS) and a Toll-like receptor-7 (TLR7) agonist (vesatolimod) for induction of ART-free HIV control.^16^ Social science research within the FRESH cohort highlighted the psychological challenges faced by study participants as they managed the requirements of the trial to pause their HIV treatment. A key learning from the trial was the importance of providing information and support to build trust with trial participants.^17^ The FRESH clinical trial and the associated studies represent an important milestone in HIV cure research, underscoring the feasibility, importance and challenges of performing HIV cure research in resource-limited urban settings. However, a significant proportion of PLWH in Africa and other LMICs live in rural settings and there is a requirement for more work to understand their knowledge, perceptions, attitudes and challenges for participation in HIV cure research and potential uptake of cure interventions when they become available.

### Building support for HIV cure research

To support the conduct of further HIV cure research in Africa, in both rural and urban settings, there is a need to build and enhance community and public understanding of HIV cure strategies and mobilise communities for locally driven initiatives. This is important not only so that the general public can understand what the term ‘HIV cure’ means and what the research may entail, but also so that the stage at which cure research has reached and the timelines for that research can be understood. Realistic expectations of the timeline to a hoped-for cure can dispel hopes of an instant cure and build trust and confidence in, as well as the public understanding of this science. Many people living with HIV live in resource-limited settings in rural parts of Africa, therefore, building an understanding of the social context of people’s lives in these settings is important to support the expansion of HIV cure research sites and nurture readiness for the potential benefits of HIV cure interventions in the future.

The need for research on the social context in which HIV cure research takes place and the general public’s understanding of HIV cure is not new.^18,19^ It has been recognised as an important bridge between the scientists conducting the research and the knowledge of those who participate in research, specifically in settings where cultural nuances may significantly impact on the understanding of HIV cure research. Introducing new and complex topics can lead to difficulties in facilitating discussion with communities, particularly in settings where the educational level in subjects such as biological sciences, particularly people in their fifties and older, may be limited.

A way to support engagement with people with differing levels of knowledge and education is to use deliberative approaches.^20^ Deliberative approaches incorporate informed discussions, structured facilitation and active participant engagement in complex topics.^20,21^ Public engagement teams have used community dialogues as platforms to allow participants where the research is being conducted to express their views and participation, which in turn helps in understanding the community perspectives.^22^ In this study, engaging with the communities as partners in this research has been the overall goal, providing scientists with an opportunity to share HIV cure science with non-scientists and to have meaningful dialogues with the communities. Deliberative approaches were then used as a method/tool within community-based participatory research (CPBR) to stimulate dialogue, gathering qualitative insights, as described in other studies.^23,24^ The CBPR framework was a guiding foundation of our broader research throughout all stages of the research process, introducing HIV cure research to our communities. Using this approach enables information on HIV cure science to be shared, building knowledge on the topic and allowing those taking part to ask questions and to break down the complexity of the science in dialogue with HIV cure scientists. Thereafter, these participants may engage and make informed decisions on whether they would participate in HIV cure intervention trials. In this way, community dialogue draws on the existing knowledge base of community members to engage with their perspectives on the issues being discussed and debated.^25^

In this paper we outline the methods we used to communicate information about HIV cure to the rural population of uMkhanyakude, in northern KwaZulu-Natal, South Africa. This work was undertaken as part of the study ‘An investigation of the public understanding of HIV cure research in uMkhanyakude District’ based at the Africa Health Research Institute (AHRI) in South Africa. The aim of this study is to explore the public understanding of HIV cure concepts and terminology to begin the process of co-designing culturally appropriate communication strategies for scientists and other stakeholders engaged in HIV cure research, beginning with members of the community who work as community engagement officers or serve on the Community Advisory Board (CAB) at AHRI.

## Materials and methods

This study was conducted in the rural uMkhanyakude district of northern KwaZulu-Natal, where HIV prevalence stands at 30% for individuals aged 15–49 years. The municipality is generally characterised by rural communities predominantly under traditional areas.^26^ This district has been heavily affected by the HIV epidemic, but also by potential barriers to research participation: >55% of the population has less than secondary school education, 19.9% of the population aged ≥20 years has no schooling, while 5.7% had attained higher education, 28.4% had completed matric^26^ and 35% live in the lowest financial stratum, with 3.9% having no income at all.^26^

Interdisciplinary teams comprising social scientists, public/community engagement officers and laboratory scientists planned the facilitation of the dialogue, as well as the data collection and data analysis methods. Social science teams facilitated the deliberative community dialogues at AHRI in December 2024 and February 2025.

An HIV cure scientist (TN) was invited to facilitate discussions on HIV cure science and ongoing research efforts. Using the CBPR concept of inform, deliberative community dialogues started with a detailed presentation on HIV cure science, highlighting the remarkable progress in ART over the years and the significant advancements that have been made, including the recent approval of long-acting injectable antivirals. The presentation further explained why an HIV cure is needed and the current scientific approaches. Research conducted in the FRESH cohort^27,28^ and its relevance to HIV cure approaches was presented, followed by discussions on current HIV cure-related strategies such as early ART initiation, latency reversal and immunotherapy.

The first deliberative dialogue was conducted with AHRI public engagement and communication officers for approximately 1 hour. The second was conducted with members of the AHRI CAB, all of whom live in the local area and have been elected by the community to represent the research population as per the AHRI internal protocol. The deliberative dialogue with the CAB members lasted 2 hours and had an isiZulu interpreter (an AHRI public engagement officer), in their own language and promoting inclusivity and understanding. We acknowledged that our positionality as scientists and researchers may have influenced participation dynamics and interpretation of the results. To reduce these effects, we encouraged members of the public engagement team as interpreters and to help in facilitation of the sessions. Refreshments were provided at the end of each community dialogue.

Following each deliberative community dialogue, a debriefing meeting was held with experienced social scientists (RSC, MM, QS) to discuss the outcomes of the discussions. Data were analysed manually using a thematic approach. Data analysis was done manually, led by the first author (RSC), and the social science research assistants (MM and QS), who collected the data. We consulted public engagement (NM) for guidance on the CAB structure and policies as background to our analysis. The coding framework was developed by RSC from the data that the entire team reviewed and used to identify emerging codes. The main codes were reviewed, discussed and agreed on by consensus for validity, and similar codes were merged to avoid duplication. Thereafter, for this analysis, a matrix coding framework was developed in MS Excel (Microsoft 365) on which coded data were manually inserted by pasting information and illustrative quotes from the discussions against the matching themes. All data were anonymised, and participants were identified only by their gender.

## Results

The deliberative community dialogue with the public engagement team included 11 females and four males. The CAB members session included 27 females and 14 males. CAB members act as community representatives, available and willing to engage households, attend meetings and work closely with AHRI staff and leaders. Most members speak isiZulu and are also fluent in English. CAB members maintain open dialogues with the community, ensuring concerns are addressed while contributing to the ethical, cultural and quality aspects of research. Through training and orientation, CAB members build capacity to advise on community norms, participant rights, risks and ethical standards, safeguarding a strong community voice in AHRI’s research. Public engagement officers are staff members employed by AHRI to address our research community needs in the studies.

Participants in both deliberative community dialogues asked many questions about the topic, highlighting the novelty of the concept of HIV cure for this population. The question-and-answer sessions were a reflection of the complexity of the HIV cure concept in this population. Participants’ questions showed their active engagement and desire to build a foundational understanding of this topic as we begin the process of co-designing culturally appropriate communication strategies in HIV cure research. While this limited an exchange of perceptions, it provided valuable insights into knowledge gaps, demonstrated trust in the dialogue process and highlighted the importance of incorporating more information in future deliberations for the broader community.

We have grouped these questions under three themes: the nature of HIV cure trials, HIV cure scientific procedures and HIV cure messaging (Table 1).

**Table 1.** Summary of key findings

Theme	Key findings
**The nature of HIV cure trials**	Participants had questions about trial procedures, risks of ART interruption, monitoring of viral load and potential impact on partners. Most were curious about eligibility criteria, recruitment equity and wanted dissemination of trial results to the community.
	Concerns included both personal safety and the broader implications of participating in the clinical trials.
**HIV scientific procedures**	Questions focused on scientific methods for eradicating HIV, including efficacy, costs, risks and how latent virus is managed.
	Participants showed varying levels of understanding of scientific terminology, indicating the need for tailored engagement.
**HIV cure messaging**	Participants were concerned about how terminology shapes expectations and influences community perceptions.
	Accurate, culturally sensitive communication, involving healthcare workers and field staff, was encouraged.
	Comparisons were made with COVID-19 vaccine messaging to illustrate potential stigma or misunderstandings.

### The nature of HIV cure trials

Participants in both dialogues questioned the process involved in HIV cure clinical trials and the risks of stopping ART. A communications officer in the public engagement team session asked: *‘Does it not equate to someone defaulting when the ART is stopped, and the virus returns?’* Questions such as this highlighted how the health education messaging on taking ART has been effective in stressing the importance of adherence; messaging on ART interruption in cure studies seemingly went against that advice and raised concerns among some participants. There were many questions about how, during an HIV cure trial, the scientists were able to monitor what was happening to the infection in the bodies of those taking part, and how one would know if the intervention was failing, and the potential clinical implications of a treatment interruption:

*Does the study check the participant’s viral load?* (male, CAB member).

 

*Can’t we develop a scan capable of detecting HIV-infected cells?* (male, CAB member).

 

*If I was taking ARVs [antiretroviral drugs], I was definitely going to be the first to take part in such a study because I would be interested in finding a solution. I remember how far we have come with HIV treatment. If someone participates in the FRESH study, do you measure their viral load? Also, when you boost the immune system, don’t other ‘things’ or co-infections potentially reactivate?* (female, CAB member).

As well as concern for the individuals taking part in the future trial, there were also concerns for their partners. Some participants were worried about the risk of passing the virus to their partners if it comes back during the analytical treatment interruptions:

*When the virus becomes detectable is a person able to pass it to the next person?* (male, CAB member).

Discussions also focused on the eligibility of clinical trial participants in HIV cure trials and who would be chosen to take part. One CAB male participant wanted to know if **‘*the eligibility criteria were for people living with HIV only?’* and another CAB member asked: *‘Does the study do prior tests before the participants enrol?’*

Underlining the hopes that rest in these trials finding a cure, others expressed a desire for equity in clinical trial recruitment:

*I wish the HIV cure study could be initiated for everybody. We learned that the previous ART treatment had a lot of side effects, and eventually, progress was made today we learned that there is an injectable treatment, and only one regimen is taken. Clinical trials are happening to help people to live a better life* (male, CAB member).

Given that the FRESH trial had been used as an example during the introduction to the concept of HIV trials, it was not surprising that participants wanted to know more about the findings from that clinical trial and stressed the importance of disseminating the results to the community. In the community engagement officers’ session, the participants were particularly interested to know what the learnings from that trial were for people like them, that is, community engagement and communications staff. These participants were also keen to understand more about the FRESH clinical trial, perhaps anticipating questions from the public as information on the findings spread. Others in the same public engagement session and some in the CAB session asked detailed questions on the trial procedures, as they sought to understand the conduct of the trial: *How many have interrupted [treatment] so far?* (public engagement officer).

*Why the study targeted women, and which precautions are followed?* (communications officer).

*As part of your study’s eligibility criteria, do you take people who are taking chronic medication?* (male, CAB member).

*How often do they have to take the reactivation drug?* (male, CAB member).

One CAB member summed up the concerns of many participants, in that people would need reassurance that the cure actually worked, given the risks involved in taking part:

*Do you have any published material that can provide proof that the HIV cure procedures work? If you’re coming to our community to conduct a study like this, we need proof or evidence that what you’re proposing will work. It’s scary to participate in such a study, especially since the concept of waking up a latent virus is concerning—what if it doesn’t go back to being latent again?* (female, CAB member).

The detailed questions asked in these sessions showed that the participants were interested and even enthusiastic about these studies; they also wanted to know not only who could take part, how they were chosen and what happened to them in the trial; and they also wanted to understand how the methods described in the introductory talk could remove HIV from someone’s body.

### HIV cure scientific procedures

There were many questions on the scientific methods to eradicate HIV, often asking for more detail on how the virus was removed:

*From the methods you have described above, what type of scientific cure can you say that completely eradicates the HIV virus?* (community engagement officer).

The CAB members generated questions regarding the cost, efficacy of the HIV cure scientific approaches and the possible risks to clinical trial participants:

*This sounds promising, but are HIV cure procedures not expensive? Could the high cost not discourage doctors from doing them for individuals with advanced HIV, such as a bone marrow transplant?* (male, CAB member).

 

*Is there a way in which you can completely clean human cells? Are human cells replaceable?* (male, CAB member).

Participants in both sessions wanted more information on the ‘analytical treatment interruption’ as a method, who it was for and the risks to participants involved in those procedures. For instance:

*Does HIV return to the blood after ART interruption?* (community engagement officer).

*Are ATI [analytical treatment interruption] studies only for participants with acute HIV?* (communications officer).

 

*Do the ATI [analytical treatment interruption] study participants understand the risks?* (communications officer).

 

*How long does HIV remain undetectable?* (male, CAB member).

 

*Does latency mean undetectable?* (male, CAB member).

Some of the questions asked showed that some participants had a good understanding of at least some of the language used in the description of the scientific procedures:

*Is there research currently being conducted on harvesting cells from people who don’t have CCR5 receptors?* (male, CAB member).

Although public engagement staff and members of the CAB may have greater knowledge of HIV science than members of the general public, the highly varied nature of the questions provided insights into the need to engage on many different levels during these sessions depending on individuals’ understanding.

### HIV cure messaging

The HIV cure messaging and the terminologies used, and the way different community members may interpret them, were of considerable interest to participants in both sessions. In the public engagement dialogue, participants were concerned about the expectations placed on this research because of the terminology used:

*Does calling HIV cure not construct hope in people that they are finally receiving cure; the expectations should be managed?* (community engagement officer).

The importance of HIV cure messaging was emphasised:

*Community engagement and field workers need to be educated about HIV cure. I suggest that health care workers also need to be involved in such a study. As they are trusted members of the community. The fieldworkers as well to help with messaging* (community engagement officer).

Parallels were drawn with the messaging around the COVID-19 vaccine, which some participants felt had not been handled well and had contributed to stigma within their communities, as well as affecting the way future research—including HIV cure research—might be received by community members:

*Our current challenge is that in the community people don’t receive the term vaccine well because of myths about COVID vaccine. We know it’s an old term but it has negative connotations now. A common belief is that vaccines kill people as we now sometimes have people sleep and wake up dead. Is there an alternative term we can use in the community?* (male, CAB member).

For some, the risks posed by HIV cure research seemed too great, with one participant urging alternative methods through which to end the HIV epidemic:

*Can’t scientists create a culture where people fear HIV, just as they fear snakes? While curing HIV is important, what kind of warnings can we use to instill fear in children to prevent them from acquiring it? What if we consider a third approach to curing HIV—focusing on educating young people? You have the power to influence people* (male, CAB member).

## Discussion

Our findings show that study participants were interested in and cautiously supportive of HIV cure research but had limited knowledge and understanding of HIV cure trials and what HIV cure scientific procedures involved. Participants were particularly concerned about the HIV cure messaging to community members. The deliberative community dialogues were intended to encourage discussions; however, because of the novelty of the concept of ‘HIV cure’ and a lack of shared understanding, the sessions became question-and-answer sessions, as participants sought to build their knowledge on the topic. While this engagement was encouraging, it was clear that those taking part wanted details about the complex science. This highlights the need to adapt and contextualise ways in which communities engage with unfamiliar or complex topics.^29,30^ Our findings are consistent and reinforce the lessons from a study exploring experiences of community dialogues between different groups within communities—health workers and local government officials in Nigeria^31^—which showed that shared decision making was hindered due to different levels of knowledge between health experts and the communities.

Our results showed that community dialogues and ongoing engagement play a vital role in the knowledge transfer for HIV cure research, as has been illustrated in other research.^32^ Deliberative community dialogues conducted in local languages are even more effective as they encourage inclusive discussions with community members.^33,34^ Community dialogues are useful in encouraging critical thinking and action planning in response to health problems.^35^ However, power inequities that arise due to expert knowledge and training as well as differences in community knowledge can lead to top-down engagement with the community.^36,37^ A deliberative dialogue of scientists with community representatives and peers who better understand the local cultural context, as we attempted here, may be one way to mitigate the risk of the top-down approach.

Our results showed that it is essential that researchers and community engagement officers have accurate and culturally sensitive information to support effective HIV cure messaging before they engage with communities. Involving public healthcare workers is equally important, as they are trusted figures within the community and can help build confidence and reduce stigma surrounding HIV cure research participation. Community dialogues can also encourage the willingness of people to participate in health research.^38^

In this study, we learnt that community engagement—particularly through the involvement of CAB members—may be one approach to communicate difficult and complex concepts to the community instead of relying on healthcare workers, who may already be overburdened. Our dialogues provided them with an opportunity to express their views and concerns regarding HIV cure research. The deliberations mobilised the CAB members and public engagement teams with knowledge to facilitate engagement with our communities as we are preparing for participant recruitment for our study on ‘An investigation of the public understanding of HIV cure research in uMkhanyakude District’. The general public, just like the public engagement team and CAB members, will have different levels of knowledge because of their educational backgrounds and their exposure to previous research or information gained from the media, and, as we learnt in these sessions, it is important to be able to explain seemingly simple concepts clearly, as well as be able to engage with those with a more detailed understanding of scientific research procedures who wish for detailed knowledge. CAB members, who are embedded within local communities, may be better positioned to build culturally sensitive dialogues, dispel misinformation and promote the uptake of services in a way that feels more accessible and less institutional, thereby enhancing the overall impact of health interventions.

Interestingly, the findings from our study were similar to those from other studies conducted in high income settings on perceptions of HIV cure research and willingness to participate in HIV cure-related trials. The same concerns, such as worrying about the risks of interrupting ART,^10,39^ the consequences of viral rebound, partner protection^40^ and limited knowledge,^41^ were raised in the setting of uMkhanyakude. In our setting, the focus has been on knowledge gaps, unlike in higher income settings, where awareness is generally higher.^41^

This study has some limitations. It would have been useful to expand this work on deliberative community dialogues to the broader population. Unfortunately, time and resources did not permit the roll out of additional dialogues in the wider community. Our community dialogues did not further explore the nature of knowledge or enhance trustworthiness by providing participants with the opportunity to engage with, add to and interpret data after these sessions because this was an ongoing research engagement. That said, we believe that the sessions we conducted provided useful insights into community members’ questions and concerns. Future studies may need to explore the effectiveness of using community representative groups instead of healthcare workers to communicate difficult, complex and novel concepts such as HIV cure research for greater societal benefit and impact.

### Conclusion

The current study highlights limitations on key knowledge around terminology and scientific approaches used in HIV cure research, particularly for practices such as analytical treatment interruption, used in clinical interventions in HIV cure research. Enthusiasm and cautious optimism for HIV cure research was expressed among community and public engagement officers and CAB members within a rural setting with a disproportionately high burden of HIV. The deliberative community dialogues were useful because they showed us that the community needed more information regarding HIV cure research. Engaging with these groups revealed the ongoing challenges in effectively communicating complex scientific concepts and highlighted the importance of ongoing community engagement and education in HIV cure research. CAB and public engagement teams not only received the information but were also eager to participate in the research moving forward. Active engagement and dialogue will require ongoing engagement and HIV cure messaging that is understandable in this setting.

## Data Availability

The deliberative dialogue transcripts cannot be publicly shared because the data contain sensitive information that could potentially identify the participants. The restriction is a guideline set by the ethics committee and included in the informed consent that participants have agreed to. Access to the data is restricted to protect participant confidentiality. Any data access requests must be reviewed and approved by the authors and will only be granted under conditions that ensure participant anonymity. Any data requests can be directed to rujeko.chidawanyika@ahri.org.
